# Supplementary material for: RORγ Structural Plasticity and Druggability
Source: Int J Mol Sci. 2020 Jul 27;21(15):5329. doi: 10.3390/ijms21155329 (PMC7432406; doi:10.3390/ijms21155329)
Supplement: Supplementary file 1 [file ijms-21-05329-s001.pdf]

Review

RORγ structural plasticity and druggability (Supplementary data)

Mian Huang[1], Shelby Bolin[2], Hannah Miller[1] and Ho Leung Ng[1*]

[1] Department of Biochemistry and Molecular Biophysics, Kansas State University
[2] Division of Biology, Kansas State University
[*] Corresponding author. Email: hng@ksu.edu

Table S1. The list of 18 co-crystals of the RORγ LBD-agonist complexes.

3KYT	3L0J	3L0L	4S14	4WPF	5APH	5IZ0	5NI8	5NIB
5NTI	5NTN	5VB7	5YP5	5YP6	6E3G	6NWU	6W9I	6W9H

Table S2. The list of 69 co-crystals of the orthosteric RORγ LBD-inverse agonist complexes.

5EJV	4NB6	5NTQ	5VB6	4QM0	4WLB	4WQP	4XT9	5AYG	4ZJW
4ZJR	4ZOM	5ETH	5IXK	5APJ	5X8X	5X8Q	5UFR	5UFO	5UHI
5NI7	5NU1	5NI5	5VB5	5NTP	5NTW	5NTK	5ZA1	6A22	5W4V
5W4R	6IVX	6J1L	6J3N	6ESN	6B33	6B30	6B31	6FGQ	3B0W
6BR2	6BR3	6FZU	6G07	6G05	6CN6	6CN5	6CVH	6Q2W	6Q6M
6Q6O	6Q7A	6Q7H	6E3E	6R7A	6R7K	6R7J	6NAD	6NWS	6NWT
6O98	6O3Z	4NIE	6P9F	6U25	5M96	6VQF	6BN6	4YMQ	

Table S3. The Classification of 69 orthosteric RORγ LBD-inverse agonist complexes.

Modes	RMSD range (Å)	Crystal models
Mode I	0 < 2	5EJV, 4NB6, 5NTQ, 5VB6, 5QM0, 4WLB, 4WQP, 4XT9, 5AYG, 4ZJW, 4ZJR, 4ZOM, 5ETH, 5IXK, 5APJ, 5UFR, 5UFO, 5UHI, 5NI7, 5NU1, 5NI5,5VB5, 5NTP, 5NTW, 5ZA1, 5W4V, 5W4R, 6J1L, 6J3N, 6ESN, 6B33,6B30, 6B31, 3B0W, 6BR2, 6BR3, 6FZU, 6G07, 6G05, 6CN6, 6CN5, 6CVH, 6Q2W, 6Q6M, 6Q6O, 6Q7A, 6Q7H, 6E3E, 6R7A, 6R7K, 6R7J, 6NAD, 6NWS, 6NWT, 6O98, 6O3Z, 4NIE, 6P9F, 6U25, 5M96, 6VQF, 6BN6, 4YMQ
Mode II	2 < 3	5X8Q, 5X8X, 6A22, 6IVX
Mode III	3 < 4	5NTK
	4 < 5	6FGQ

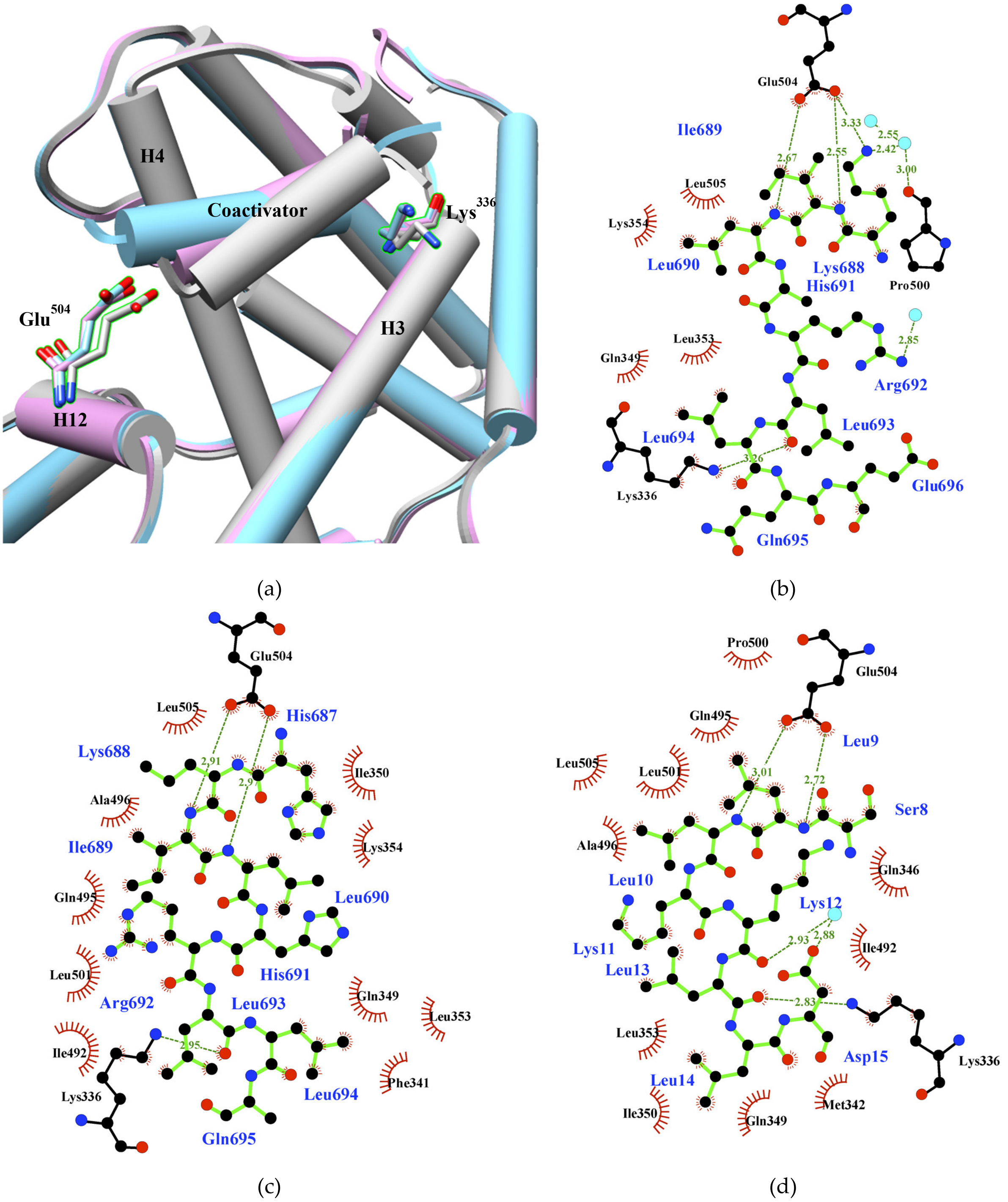

Figure S1. Atomic interactions between the coactivators and RORγ LBD unbound or bound with orthosteric inverse agonists. (a) The crystal structures of the apo LBD (grey) and two ligand-bound LBDs (PDB 4NIE, cyan; PDB 4WLB, plum) are aligned. The coactivators bind to the LBD surface in different ways. The 2D interaction diagrams are presented using LigPlot+ [1] with coactivators in green sticks, hydrogen-bonding residues in black sticks and hydrophobically interacting residues in red eyelash shape. The non-covalent interactions between the coactivators and the surrounding environment are diverse among the unbound form (b) and the bound forms (c for 4NIE and d for 4WLB).

Protocol S1. Homology model preparation and molecular docking

The RORγ full-length homology models were built using the default settings of I-TASSER online server. The top five predicted models are output, accompanied with the quality evaluation for each model (Table S4) and the normalized B factor for the best one (Figure S2).

Table S4. Evaluations of homology models predicted by I-TASSER.

	Model 1	Model 2	Model 3	Model 4	Model 5
C-score	-2.24	-2.42	-2.58	-2.87	-3.09
Estimated TM-score	0.45±0.15	--	--	--	--
Cα RMSD to Model 1	0.0000	16.8002	12.9024	31.1794	7.3447
LBD Cα RMSD *	5.0627	5.3387	5.5153	13.0577	5.0643

* The LBD of every model was compared with the apo LBD crystal model (PDB 5X8U).

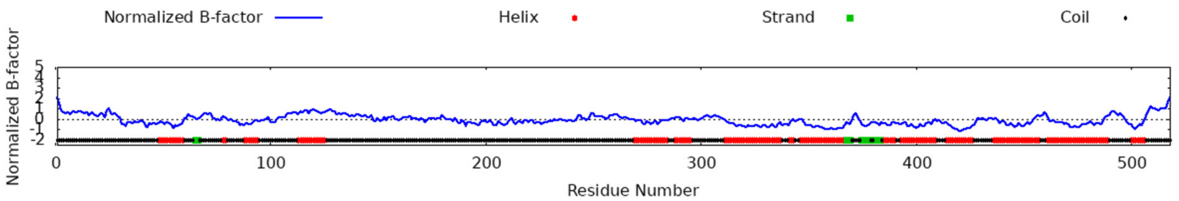

Figure S2. Predicted normalized B factor of the top homology model 1 (purple in Figure 9a).

We chose model 1 for molecular docking in YASARA, due to its similar shape to the structure published by Lao et al. Compound **10** was energy-minimized with the AMBER14IPQ force field [2] and then docked against the whole model 1 using the default settings of AutoDock Vina [3]. To search other potential binding modes, we also docked **10** against the whole model 3 (tan in Figure 9a) and model 5 (salmon in Figure 9a) by the same method. Models 2 and 4 were not considered here because the LBDs and/or DBDs in the models are partially disordered leaving inadequate space for the HD between them. At least three independent docking experiments were executed for each model, and the top-ranked binding pose(s) located in the HD site with the highest binding energy (Table S5) were picked.

Table S5. The performance of compound **10** in different docking experiments

Model	Ranking (out of 50)	Binding energy (kcal/mol) *	Corresponding to
1	3	7.45	Figure 9b
3	14	7.70	Figure 9c
5	8	7.27	Figure 9d
5	10	7.12	Figure 9e

* YASARA defines that the more positive the binding energy, the more favorable the interaction.

Co-crystal models of the LBD bound with orthosteric inverse agonists were selected for molecular docking. The models were firstly prepared by removing ligands, water molecules and solvent ions. Then **10** was docked against each model by following the docking method mentioned above. Besides from the one shown in Figure 9f, the docking results of other models are displayed in Figure S3. All of them are ranked in the first position of the docking results with binding energy calculated. The binding energy of the one in Figure 9f is 10.02 kcal/mol.

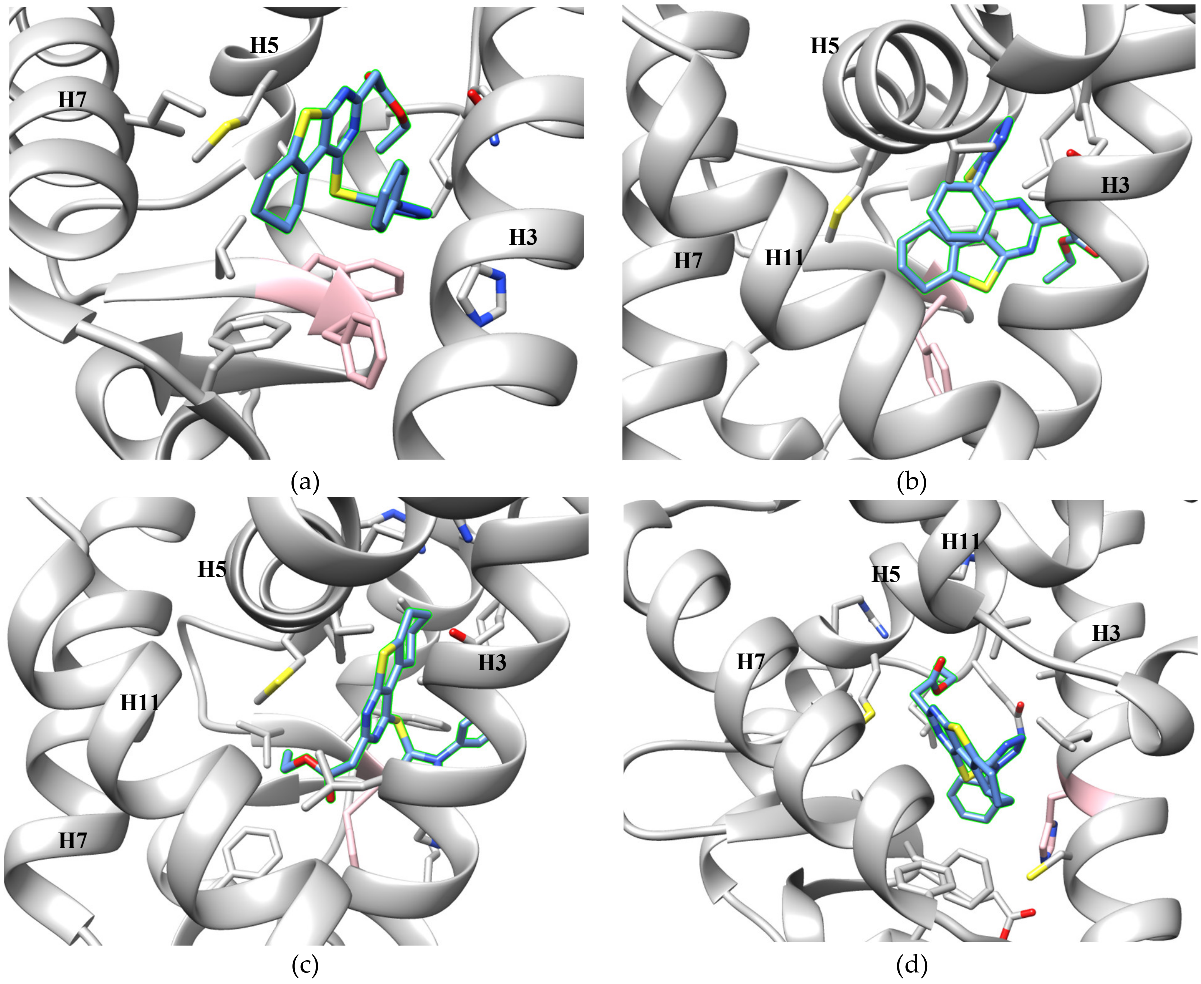

Figure S3. The docking poses of compound **10** in the orthosteric pocket of RORγ. The residues potentially interacting with **10** (blue) through hydrophobic effects and π stacking are marked in grey and pink colors, respectively. The crystal models and binding energy calculated by YASARA are listed as follows: (a) PDB 3B0W, 9.32 kcal/mol; (b) PDB 5X8Q, 8.34 kcal/mol; (c) PDB 6IVX, 9.35 kcal/mol; (d) PDB 5NTK, 9.15 kcal/mol.

References

1. Laskowski, R.A.; Swindells, M.B. LigPlot+: multiple ligand-protein interaction diagrams for drug discovery. *J. Chem. Inf. Model.* **2011**, *51*, 2778–2786, doi:10.1021/ci200227u.
2. Cerutti, D.S.; Swope, W.C.; Rice, J.E.; Case, D.A. ff14ipq: A Self-Consistent Force Field for Condensed-Phase Simulations of Proteins. *J. Chem. Theory Comput.* **2014**, *10*, 4515–4534, doi:10.1021/ct500643c.
3. Trott, O.; Olson, A.J. AutoDock Vina: Improving the speed and accuracy of docking with a new scoring function, efficient optimization, and multithreading. *J. Comput. Chem.* **2010**, *31*, 455–461, doi:10.1002/jcc.21334.
